# High-dose chemotherapy in male germ cell cancer patients—a study by the SWENOTECA group

**DOI:** 10.1038/s41416-025-03322-9

**Published:** 2025-12-22

**Authors:** Anna K. Jansson, Gabriella Cohn-Cedermark, Helene F. S. Negaard, Torgrim Tandstad, Olof Ståhl, Annika Hedlund, Åsa Karlsdottir, Martin Hellström, Carl W. Langberg, Camilla Sköld, Hege S. Haugnes, Ingrid Glimelius

**Affiliations:** 1 Department of Immunology, Genetics & Pathology, Cancer Precision Medicine, Uppsala University, Uppsala, Sweden; 2 Department of Oncology-Pathology, Karolinska Institutet, Stockholm, Sweden; 3 Department of Pelvic Cancer, Genitourinary Oncology Unit, Karolinska University Hospital, Stockholm, Sweden; 4 Department of Oncology, Oslo University Hospital, Oslo, Norway; 5 The Cancer Clinic, St. Olavs University Hospital, Trondheim, Norway; 6 Department of Clinical and Molecular Medicine, NTNU, Trondheim, Norway; 7 Department of Oncology, Skåne University Hospital, Lund, Sweden; 8 Department of Oncology, Sahlgrenska University Hospital, Gothenburg, Sweden; 9 Department of Oncology, Haukeland University Hospital, Bergen, Norway; 10 Department of Diagnostics and Intervention, Oncology, Umeå University, Umeå, Sweden; 11 Department of Oncology, University Hospital of North Norway, Tromsø, Norway; 12 Institute of Clinical Medicine, University of Tromsø, Tromsø, Norway

**Keywords:** Germ cell tumours, Outcomes research, Testicular cancer

## Abstract

**Background:**

World-wide consensus is lacking regarding patient selection and timing of high-dose chemotherapy (HDCT) with autologous stem-cell support in males with germ cell cancer (GCC). However, within the Swedish and Norwegian Testicular Cancer Group (SWENOTECA) guidelines are harmonised. Our aim was to evaluate survival and toxicity in HDCT-treated GCC-patients within SWENOTECA.

**Methods:**

In total, 7322 GCC patients were diagnosed between 2011–2021 in Sweden and Norway, among which 80 ( ~ 1.1%) patients were treated with HDCT. Indications for HDCT were: Delayed tumour-marker decline during primary/intensified primary treatment (*n* = 45), progressive disease (*n* = 29), or relapse (*n* = 25). The HDCT-regimen consisted of two cycles of carboplatin/etoposide.

**Results:**

The 5-year overall survival (5-yr OS) and progression-free survival (5-yr PFS) after HDCT was 55% and 43%, respectively. Indication delayed tumour-marker decline: 5-yr OS:75%, 5-yr PFS:53%, indication progressive disease 5-yr OS:29%, 5-yr PFS:18%, indication relapse 5-yr OS:61%, 5-yr PFS:58%. Four (5%) died due to HDCT-related complications, none within the delayed tumour-marker decline group. Grade 3–4 infections were seen in *n* = 69, 86%.

**Conclusion:**

HDCT-treatment according to the SWENOTECA-programme was feasible and associated with promising OS. Early intensification to HDCT for patients with delayed tumour-marker decline during primary-treatment was safe and led to 5-yr OS of 75% with no toxic deaths.

## Introduction

Germ cell cancer (GCC) is the most common malignancy among young men [1], with an increasing incidence in Europe and several other parts of the world [2, 3]. Since the introduction of cisplatin-based combination chemotherapy (CBCT), the survival has improved remarkably, also for patients with an extensively metastasised disease at diagnosis [4]. The first-line treatment for metastasised GCC is standard-dose CBCT most commonly combined with bleomycin and etoposide (BEP). Patients with testicular GCC in Sweden and Norway have an overall 5-year survival of 98% [5, 6], but some patients with advanced GCC have poorer prognosis.

High-dose chemotherapy (HDCT) with stem cell support is an alternative for patients with severe GCC and/or insufficient response to previous treatment. However, due to the rarity of GCC and the generally good prognosis, randomised studies are few and consensus is lacking regarding the patient selection and timing of HDCT. Studies have failed to show a survival benefit with HDCT as first-line treatment [7–9]. However, a phase III randomised trial by Motzer et al. showed a significant benefit of HDCT in IGCCCG intermediate and poor risk GCC with delayed tumour marker decline after 2 cycles of BEP as primary treatment [9]. Furthermore, large retrospective studies, along with phase I and II trials, suggest a survival advantage of HDCT versus standard-dose chemotherapy in the salvage setting [10–14].

The Swedish and Norwegian Testicular Cancer Group (SWENOTECA) is a collaboration aiming to standardise and improve cancer treatment and care for patients with testicular and extragonadal GCC in Sweden and Norway [15]. In the SWENOTECA cancer care programmes, tumour marker decline of tumour markers human chorionic gonadotropin (hCG) and alpha-fetoprotein (AFP) has been used to stratify patients to treatment intensification with HDCT since 1995. This is a unique approach, not seen in other guidelines. In the SWENOTECA VIII guidelines from 2012, HDCT has been recommended for patients with delayed tumour marker decline during primary or intensified primary treatment, as well as for selected patients with progressive disease during, or relapse after, primary treatment for metastatic GCC.

The primary aim of this study was to evaluate survival and toxicity in patients treated with HDCT within the population-based SWENOTECA cancer care programme in 2011–2021, including patients treated with HDCT as early intensification during primary treatment.

## Methods

### Patients

All male patients with testicular or extragonadal GCC (excluding patients with intracranial primary tumour site) treated with HDCT in Sweden and Norway between 2011–2021 were included. Patients and patient data were identified from the SWENOTECA registry [15], which is a population-based registry with prospectively collected data. Completeness of the SWENOTECA registry was ensured by cross-checking with the Swedish National Cancer Registry and with each treatment centres’ database.

Patients received HDCT due to three different indications:

1. Delayed tumour marker decline during primary or intensified primary treatment (hCG T_½_ > 3 days, AFP_½_ > 7 days) (Fig. S1a).
  For treatment in the salvage setting, patients were divided into two different groups:
2. Progressive disease during primary treatment. This was defined as progression during primary treatment or within three months from last cycle of chemotherapy, without previous complete response radiologically and biochemically. This group was further divided into:
  - Patients with progression during or within four weeks of last cycle of chemotherapy (defined as cisplatin-refractory)
  - Patients with progression later than four weeks but within three months from last cycle of chemotherapy (considered cisplatin-sensitive).
3. Relapse after intensive first-line treatment, either as first-line relapse treatment for patients with intermediate or high/very high-risk according to the International Prognostic Factors Study Group (IPFSG) criteria [16], or due to poor response to, or progressive disease during, relapse treatment for patients with low risk or very low risk according to IPFSG. Relapse was defined as relapse after a complete response to previous first-line treatment and/or relapse later than three months after last cycle of chemotherapy.

Patients with progressive disease in the brain only, during or within four weeks of chemotherapy, were not considered cisplatin refractory since the brain is regarded as a sanctuary site for GCC cells with regard to chemotherapy.

The SWENOTECA cancer care programmes are since 1995 designed for early identification of patients with poor response to standard cisplatin-based chemotherapy, reported previously by Olofsson et al. [17]. In the SWENOTECA cancer care programme VIII (2012–2020) non-seminoma patients with delayed tumour marker decline were treated according to the flow-charts depicted in Fig. S1a and S1b. In summary, non-seminoma patients with delayed tumour marker response to two cycles of BEP were recommended two cycles of BEP combined with ifosfamide (BEP-if) or two cycles of cisplatin, etoposide and ifosfamide (PEI), for intermediate prognosis patients and poor prognosis patients due to markers only (no non-pulmonary visceral metastasis); or two cycles of paclitaxel, ifosfamide and cisplatin (TIP) for poor prognosis patients with non-pulmonary visceral metastasis. Stem cell harvest was performed between the first and the second cycle. Patients with contraindications to bleomycin and patients with brain metastasis at diagnosis were recommended to start treatment with PEI instead of BEP. Patients with continuous delayed tumour marker response after intensified primary treatment proceeded to two cycles of HDCT. Two patients in 2021 were treated according to an updated version of the guidelines, SWENOTECA X, proceeding to HDCT treatment directly after 1 TIP, after a delayed tumour marker decline response to two cycles of PEI.

Patients with relapse were treated according to the flow-chart depicted in Fig. S1c.

The patient inclusion is described in Fig. S2.

### HDCT treatment

Two cycles of HDCT were planned for all patients. A minimum of 7 × 10^6^ CD34+ cells/kg bodyweight in total were harvested. The HDCT cycle consisted of etoposide 560 mg/m^2^ day 1-4 and carboplatin 8 x (glomerular filtration rate + 25) mg day 1-4 (Fig. S3). Autologous stem cells were infused intravenously approximately 72 h after end of chemotherapy. The second cycle HDCT was scheduled to start as soon as the patient recovered, usually within 6-8 weeks after start of the first cycle. Patients received supportive care during HDCT according to each institution’s routines. Toxicity was assessed as grade 3–4 toxicity according to WHO 1979 [18]. Ototoxicity was assessed as documented hearing loss or tinnitus at any follow-up until one year after HDCT. Death due to HDCT was defined as treatment-related death within three months after HDCT, as reported by the local clinician in charge.

### Statistics

Continuous variables were summarised using median and range, and absolute and relative frequencies were used to present categorical variables. Overall survival (OS) was calculated from the beginning of HDCT to the date of death or last follow-up. Progression-free survival (PFS) was calculated from the beginning of HDCT to the date of progression, relapse, death or last follow-up; whichever occurred first. Observation time was calculated from the beginning of HDCT to last follow-up. Overall median follow-up was calculated using the inverse Kaplan-Meier method. Survival rates were analysed using the Kaplan–Meier method. Survival endpoints were also analysed by multivariable Cox Proportional Hazard regression, presented with hazard ratios (HR) and 95% confidence intervals (CI) and survival was adjusted for age as a continuous variable, site of primary tumour, indication and cisplatin sensitivity. The proportional hazards assumption was checked and verified for all Cox regression models using both graphical diagnostics and statistical tests. Differences in proportion was tested with Wilcoxon–Mann–Whitney test, Chi-square-test and Fisher’s exact test. Tests were performed using two-sided *p*-values with a significance level of 0.05. Statistical analyses were performed using R (version 4.2.1, R Core Team 2022).

## Results

### Patient and treatment characteristics

From the Swedish and Norwegian cancer registries, we identify 7322 GCC patients diagnosed 2011–2021. Overall, 80 patients were treated with HDCT and included in this study, of whom 76 patients (95%) were diagnosed with non-seminoma and four with seminoma (5%) (Table 1). The primary tumour site was testis in 60 patients (75%). All mediastinal primary GCC were non-seminomas. The median follow-up time was two years (range 0–11). The median follow-up time for surviving patients was five years and using the inverse Kaplan–Meier method, the overall median follow-up was 5.3 years. 24 patients received HDCT in 2020 or 2021 and therefore the follow-up of these patients are shorter.

**Table 1:** Patient characteristics by indication for high-dose chemotherapy (HDCT).

Characteristics	All patients, *N* (%)	Delayed marker decline, *N* (%)	Progression^a^, *N* (%)	Relapse, *N* (%)
*N* (% of all)	80 (100)	26 (33)	29 (36)	25 (31)
Age at treatment, years				
Age, median (range)	34 (17–63)	35 (17–63)	32 (21–60)	34 (18–46)
Patients <40 years	59 (74)	18 (69)	19 (66)	22 (88)
Patients ≥40 years	21 (26)	8 (31)	10 (34)	3 (12)
Observation time, years				
Median (range)	2 (0–11)	4 (0.6–11)	1 (0–11)	4 (0–11)
Histological type				
Seminoma	4 (5)	0	1 (3)	3 (12)
Non-seminoma	76 (95)	26 (100)	28 (97)	22 (88)
Location of primary tumour				
Testicular	60 (75)	19 (73)	19 (66)	22 (88)
Extragonadal	20 (25)	7 (27)	10 (34)	3 (12)
- Retroperitoneal	9 (11)	3 (12)	6 (21)	0 (0)
- Mediastinal	10 (13)	3 (12)	4 (14)	3 (12)
- Other	1 (1)	1 (4)	0 (0)	0 (0)
Risk group at initial diagnosis				
Good	12 (15)	1 (4)	1 (3)	10 (40)
Intermediate	11 (14)	2 (8)	4 (14)	5 (20)
Poor	57 (71)	23 (88)	24 (83)	10 (40)
Stage at initial diagnosis^b^				
CS 1	0 (0)	0 (0)	0 (0)	0 (0)
CS 2	12 (15)	1 (4)	2 (7)	9 (36)
CS 3	4 (5)	1 (4)	1 (3)	2 (8)
CS 4	64 (80)	24 (92)	26 (90)	14 (56)
Initial metastatic sites				
Lymph nodes	71 (88)	23 (88)	25 (86)	23 (92)
Lung	53 (66)	21 (81)	21 (72)	11 (44)
Liver	29 (36)	13 (50)	12 (41)	4 (16)
Brain	19 (24)	9 (35)	9 (31)	1 (4)
Bone	13 (16)	5 (19)	5 (17)	3 (12)
Other	8 (10)	3 (12)	3 (10)	3 (12)
Cisplatin sensitivity^c^				
Sensitive	64 (80)	26 (100)	15 (52)	23 (92)
Refractory	16 (20)	0 (0)	14 (48)	2 (8)
Tumour marker at start of HDCT^d^^,e^				
Not elevated	17 (21)	3 (12)	4 (14)	10 (40)
Elevated	62 (78)	23 (88)	24 (86)	14 (56)
Previous chemotherapy regimen				
BEP	50 (63)	13 (50)	15 (52)	22 (88)
BEP-if/PEI	46 (58)	17 (65)	21 (73)	8 (32)
TIP	70 (88)	20 (77)	26 (90)	24 (96)
Other	4 (5)	0 (0)	2 (7)	2 (8)
Cycles prior to HDCT, median				
Cycles, median (range)	5 (2–13)	4 (2–6)	5 (3–9)	6 (4–13)
Status				
Alive	44 (55)	18 (69)	9 (69)	17 (68)
Deceased, cause of death	36 (45)	8 (31)	20 (31)	8 (32)
*Testicular cancer*	29 (36)	6 (23)	18 (62)	5 (20)
*Side effects of HDCT*	4 (5)	0 (0)	2 (7)	2 (7)
*Other causes*	2 (3)	2 (8)	0 (0)	0 (0)

*N* Number, *HDCT* High-dose chemotherapy treatment, *CS* Clinical stage, *BEP* Bleomycin, Etoposide, Cisplatin, *BEP-if* Bleomycin, Etoposide, Cisplatin, Ifosfamide, *PEI* Cisplatin, Etoposide, Ifosfamide, *TIP* Paclitaxel, Ifosfamide, Cisplatin.

^a^Progression: Progression within 3 months from last chemotherapy treatment.

^b^Stage according to Royal Marsden, modified.

^c^Cisplatin refractory: Progression within 4 weeks from last chemotherapy treatment.

^d^Tumour markers: human chorionic gonadotropin (hCG) and alpha-fetoprotein (AFP).

^e^Tumour marker at start of HDCT is missing for one patient.

The majority of patients (*n* = 57, 71%) were classified as poor prognosis at initial diagnosis. In total, 44 patients (55%) had liver, bone and/or brain metastasis at initial diagnosis.

Overall 26 patients (33%) received HDCT due to delayed tumour marker decline and 29 (36%) because of progressive disease. Twenty-five (31%) patients received HDCT as part of relapse treatment (Table 1). Two patients relapsed within three months, but were reported as relapses since they were in complete remission radiologically and biochemically after primary treatment. Four patients relapsed more than two years after primary treatment.

Overall 56 (70%) patients received both intended HDCT cycles. The main reasons for receiving only one HDCT cycle were toxicity or progression (Table S1).

### Survival

For all patients, the 5-year OS after HDCT was 55%, and 5-year PFS after HDCT was 43% (Table 2).

**Table 2:** Overall survival and progression-free survival at 1, 2 and 5 years, all patients.

Indication for HDCT	Patients (*N*)	Deaths (*N*)	1-year OS probability	2-year OS probability	5-year OS probability
*Overall*	80	36	70 (60–81)	62 (52–73)	55 (44–68)
Delayed marker decline	26	8	88 (76–100)	80 (66–97)	75 (60–95)
Progression	29	20	48 (33–70)	33 (19–56)	29 (16–52)
Relapse	25	8	76 (61–95)	76 (61–95)	61 (41–91)
	Patients (*N*)	Events (*N*)	1-year PFS probability	2-year PFS probability	5-year PFS probability
*Overall*	80	43	50 (40–63)	46 (36–59)	43 (33–56)
Delayed marker decline	26	12	57 (41–80)	53 (37–77)	53 (37–77)
Progression	29	22	23 (11–46)	18 (8–42)	18 (8–42)
Relapse	25	9	74 (58–94)	70 (53–91)	58 (41–84)

*OS* Overall survival, *PFS* Progression-free survival.

Survival probability %; (95% CI).

**Fig. 1:**
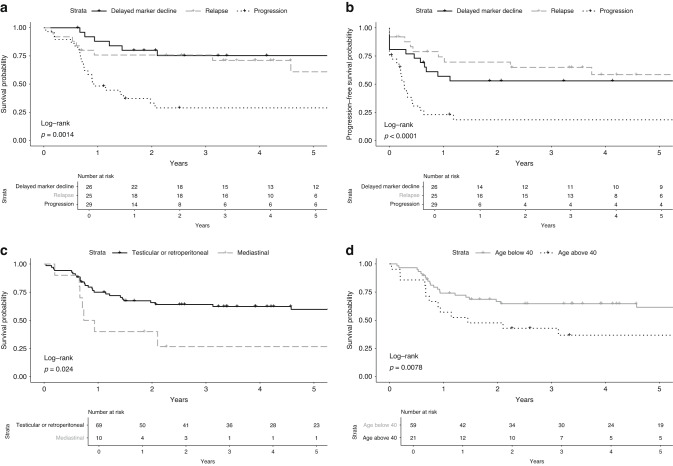
Overall survival and progression-free survival in patients treated with high-dose chemotherapy. **a** Overall survival by indication for high-dose chemotherapy. **b** Progression-free survival by indication for high-dose chemotherapy. **c** Overall survival by primary tumour site. *One individual with other location of extragonadal tumour was removed from the analysis. **d** Overall survival by age at diagnosis (years).

Patients treated with HDCT due to delayed tumour marker decline had a 5-year OS of 75% and 5-year PFS of 53%. Patients treated with HDCT due to relapse had a 5-year OS of 61% and 5-year PFS of 58%. Patients treated with HDCT due to progressive disease during primary treatment had a 5-year OS of 29% and 5-year PFS of 18% (Fig. 1a, Table 2). Among the patients treated with HDCT due to progressive disease, 14 (48%) were deemed cisplatin-refractory; these patients had a 5-year OS of 21% and a 5-year PFS of 14% (Fig. 1a–b).

Patients who were treated with HDCT due to progressive disease had significantly worse OS than patients who were treated with HDCT due to delayed tumour marker decline or relapse (HR 3.50; 95% CI 1.53–7.99). This difference persisted after adjusting for age, primary tumour site and cisplatin sensitivity (HR 4.61; 95% CI 1.67–12.65) (Table 3).

**Table 3:** Univariable and multivariable Cox regression analyses with hazard ratio (HR) for all-cause mortality.

	Univariate HR (95% CI)	Adjusted HR (95% CI)*
Age (continuous variable)	**1.04 (1.01–1.07)**	**1.04 (1.00–1.08)**
< 40 years old	Ref	-
> 40 years old	**2.40 (1.24–4.67)**	**-**
Site of primary tumour^a^		
Testicular or retroperitoneal	Ref	Ref
Mediastinal	**2.57 (1.11–5.97)**	2.32 (0.90–5.96)
Indication for HDCT^a^		
Delayed marker decline	Ref	Ref
Progression	**3.50 (1.53–7.99)**	**4.61 (1.67–12.65)**
Relapse	1.20 (0.45–3.21)	1.69 (0.58–4.92)
Cisplatin sensitivity		
Cisplatin sensitive	Ref	Ref
Cisplatin refractory^b^	**2.54 (1.24–5.21)**	1.26 (0.55–2.92)
Marker at start of HDCT		
Positive	Ref	-
Negative	0.71 (0.30–1.71)	-
Cycles of chemotherapy		
Number of chemotherapy cycles	0.99 (0.82–1.18)	-
< 5 cycles	Ref	-
5–6 cycles	1.00 (0.47–2.14)	**-**
> 6 cycles	1.39 (0.52–3.72)	-

*HR* hazard ratio, *HDCT* high-dose chemotherapy, *CI* confidence interval, *Ref* Reference group.

Significant associations are indicated in bold.

^a^Significant variables in the univariate model were included in the multivariable model.

^b^Cisplatin refractory: Progression within 4 weeks from last chemotherapy treatment.

Patients with primary mediastinal extragonadal GCC had a 5-year OS of 27%, while patients with primary GCC in the testicle or retroperitoneum had a 5-year OS of 60% (Fig. 1c). Patients with primary mediastinal GCC had significantly worse outcomes (HR 2.57; 95% CI 1.11–5.97), however this did not remain statistically significant when adjusting for age, indication and cisplatin sensitivity (HR 2.32; 95% CI 0.90–5.96) (Table 3).

Older age at diagnosis was associated with a worse prognosis (HR 1.04;95% CI 1.00–1.08 per year, adjusted, *p* = 0.039) (Table 3) and patients older than 40 years had significantly poorer survival (*p* = 0.008) (Fig. 1d).

### Patients with long-term survival despite progression after HDCT

Of the 44 patients that relapsed after HDCT, 11 patients (14% of all patients) became long term disease-free survivors at last follow up with additional salvage treatment, with a median follow-up after HDCT of 3.4 years (range 1.5–11.1 years). The majority of these patients were treated with EMA-CO (etoposide, dactinomycin, methotrexate, cyklofosfamide and vincristine), alone or combined with surgery and/or radiation therapy. The remaining patients were treated with cabazitaxel, GOP (gemcitabine, oxaliplatin, paclitaxel) or TIP combined with radiation therapy and/or surgery, or with surgery only.

### Toxicity

The majority of patients (86%) were treated for grade 3–4 infections, including febrile neutropenia (Table 4). Four patients (5%) died due to HDCT toxicity.

**Table 4:** Toxicity of high-dose chemotherapy.

	High dose cycle 1	High-dose cycle 2	
Number of patients (*N*) (% of all)	80 (100)	56 (70)	
Days to ANC^a^ > 1.0 × 10^9^/L	16 (11–49)^1^	16 (9–20)^3^	
Days to platelet count > 20 × 109/L	18 (12–52)^2^	17 (9–44)^3^	
Days of inpatient care, median (range)	22 (8–70)	19 (8–100)^4^	
*Toxicity*	High dose cycle 1, *N* (%)	High-dose cycle 2, *N* (%)	Overall, *N* (%)
Number of patients (*N*)	80 (100)	56 (100)	80 (100)
Infection including febrile neutropenia^a^	63 (79)	46 (82)	69 (86)
Gastrointestinal toxicity^b^	19 (24)	8 (14)	23 (29)
Ototoxicity^c^	NA	NA	21 (26)^5^
Neurotoxicity^a^	7 (9)	9 (16)	13 (16)
Nephrotoxicity^a^	6 (8)	4 (7)	10 (13)
Cardiac toxicity^a^	3 (4)	5 (9)	7 (9)
Bleeding^a^	3 (4)	3 (5)	6 (8)
Pulmonary toxicity^a^	2 (3)	2 (5)	4 (5)
Liver toxicity^a^	2 (3)	2 (5)	4 (5)
Other			
Any toxicity	68 (85)	43 (77)	71 (89)
Death due to HDCT	1 (1)	3 (5)	4 (5)

*ANC* Absolut Neutrophil Count, *NA* not available, *HDCT* High-dose chemotherapy treatment

Toxicities are listed in order of frequency.

^1^Missing information: 9 patients, ^2^ Missing information: 8 patients, ^3^ Missing information: 5 patients, ^4^ Missing information: 1 patient, ^5^Missing information: 2 patients.

^a^Grade 3-4 according to WHO 1979.

^b^Colitis, perforation, ischaemia, ileus.

^c^Documented hearing loss or tinnitus grade unspecified (UNS) at any follow-up up until one year after high dose chemotherapy (HDCT).

## Discussion

We show in a unique study a 5-year OS of 55% and a 5-year PFS of 43% for patients with advanced GCC who have been treated with HDCT with autologous stem cell support according to the SWENOTECA cancer care programme between 2011–2021 in Sweden and Norway. We also present 5-year OS results of 75% for patients treated with HDCT as early intensification due to delayed marker decline, with no treatment-related deaths in this group, providing a promising approach for high-risk patients on a population-based bi-national level.

### HDCT as early intensification

Although phase II studies on HDCT in GCC have shown promising results [19–21], the three randomised studies exploring HDCT as first-line treatment in poor prognosis GCC have failed to show any survival benefit compared to standard-dose chemotherapy [7–9]. However, one study closed early due to slow accrual with subsequent low power [7], and the other used substantially lower doses of chemotherapy than in contemporary HDCT regimens [8]. In the third study, a phase III study by Motzer et al, patients with delayed tumour marker decline after two cycles of BEP subsequently treated with two cycles of HDCT had significant improved complete response proportion and a trend towards better survival (2-year survival rate of 78% versus 55%; *p* = 0.1) compared to those treated with BEP only [9]. In this study, we have been able to reproduce these results in a real-world setting and shown that patients with delayed tumour marker decline transitioned to intensification with HDCT have even slightly higher 2-year survival rates than in the Motzer trial; 80% versus 78%.

In the present study, patients with delayed tumour marker decline received four cycles of chemotherapy prior to the HDCT, while patients in the trial by Motzer et al. only received two cycles of chemotherapy. The rationale for this was that patients with adequate tumour marker decline during intensified primary treatment did not have to proceed to HDCT. However, after a revision of the guidelines in 2021, patients with the most advanced disease, poor prognosis due to non-pulmonary visceral metastases who have delayed tumour marker decline after two cycles of primary treatment (BEP or PEI), now proceed directly to HDCT.

A majority of patients (88%, *n* = 23) treated with HDCT due to delayed tumour marker decline in this study had poor risk group GCC. Delayed tumour marker decline is established as a negative prognostic factor [22, 23]. The 5-year OS for our patients treated with HDCT due to delayed tumour marker decline was 75%, i.e., comparable to the 5-year survival of the poor risk group overall (73% in the SWENOTECA registry in Sweden between 1995–2022 [5] and better than the described 5-year OS of 67% according to the IGCCCG [24]). Although it is not possible to draw significant conclusions due to few patients in this study and lack of randomised control group, this would indicate that HDCT increases survival rates for this group up to the survival rates in patients without delayed tumour marker decline.

### HDCT as salvage treatment

In addition, there is no clear consensus internationally regarding the place for HDCT treatment in the salvage treatment [25–27]. In SWENOTECA guidelines, HDCT is recommended as first-line salvage treatment to patients with IPFSG-score [16] intermediate risk or worse, or IPFSG-score low risk or better with delayed tumour marker decline.

Phase I and phase II studies, along with large retrospective series, have shown an advantage of HDCT in the salvage setting, recently assessed as favourable in a review by Chovanec et al. [10].

In the largest retrospective study from 2011 [11], Lorch et al. showed that HDCT was superior as first-line salvage treatment compared to standard-dose in all risk-groups apart from the low-risk group. The study excluded patients with platinum-refractory disease. The 5-year OS in patients treated with HDCT with intermediate, high-risk and very high-risk was 58%, 35% and 27% respectively. These results are in line with our results with a 5-year OS of 61% for patients treated due to relapse, and a 5-year OS of 29% for patients treated due to progressive disease.

Patients with progression within three months of primary chemotherapy in our study had a strikingly short 5-year OS when compared to patients treated due to relapse after a complete response or with a relapse later than three months after primary treatment. Progression within three months from initial chemotherapy is known as a negative prognostic factor [16]. The relatively high proportion of patients with progressive disease in the present study (36%) may affect comparisons to other studies. Furthermore, patients in the present study had a high percentage of poor prognosis at initial diagnosis (71%), brain, bone or liver metastasis at initial diagnosis (55%), a high percentage of patients had mediastinal primary GCC (13%), and there were few seminoma patients (5%) compared to other studies [11, 13, 14]. This reflects that the patients selected to HDCT according to the SWENOTECA guidelines constitutes a high-risk group.

### HDCT for patients with mediastinum as primary site

Patients with mediastinal primary GCC have a poor prognosis according to IGCCCG [28]. The two-year PFS in patients with mediastinal primary GCC was 30% in our study, indicating that some patients with mediastinal primary tumour seem to benefit from HDCT treatment. However, of the four patients with mediastinal primary GCC treated with HDCT due to progression, all had a PFS of less than six months and an OS of less than a year making it a less hopeful option.

### Toxicity

The toxicity of HDCT treatment in this study was in line with or in some cases higher than in other studies [7–9, 13, 14, 21]. Four patients (5%) died due to HDCT treatment, which is comparable to several previous studies [8, 9, 29], although some studies have reported treatment-related mortality to be as low as 1.6–2.5% [13, 14]. There were no deaths within the delayed tumour marker decline group. The reporting of mortality was rigorous in our study and in Sweden and Norway 100% of all deaths are registered due to availability of nationwide register-based data.

### Strengths and limitations

This study is population-based, including all patients treated with HDCT in Sweden and Norway during the study period, which is a strength of this study, along with the prospectively collected data. Limitations of this study is the relatively small sample size, affecting power in the subgroup analysis, and short follow-up for some of the patients. Despite this, this study sheds further light on the usage and advantages of HDCT in patients with advanced GCC, with detailed data on treatment and outcome for all patients. The ongoing TIGER trial [30], randomising poor prognosis GCC patients to either HDCT or TIP as first-line salvage treatment, may give us better understanding of the benefits of HDCT treatment in the salvage setting in the future. Furthermore, maintenance oral etoposide after HDCT have showed encouraging results in previous studies [31], and a randomised phase II trial investigating this is also ongoing [32].

There is an urgent need for new treatments for patients with poor response to HDCT treatment. In our study, 11 patients were successfully treated with other regimens following relapses after HDCT treatment, the most common successful treatment being EMA-CO with or without combination with surgery and/or radiation therapy. EMA-CO is in SWENOTECA guidelines reserved for patients with a human chorionic gonadotropin-producing tumour and/or known choriocarcinoma histology. There are ongoing studies exploring new treatments, such as chimeric antigen receptor T cell therapy for claudin-6 positive GCC patients [33]. Although these results are highly anticipated, HDCT will have a remaining role in the treatment of patients with advanced GCCs, and further studies are needed to evaluate and fine-tune which patients benefit from HDCT.

## Conclusion

In conclusion, we have shown that HDCT for patients with advanced GCC is feasible according to the SWENOTECA strategy, and leads to 5-year OS and PFS rates of 55% and 43% respectively. The approach with early intensification to HDCT for patients not responding adequately to primary treatment is safe, leads to favourable OS and PFS rates and may be advantageous. Finally, even though patients that receive HDCT due to progressive disease have relatively poor outcomes, 29% of patients did achieve long-term survival, and this should still be considered in lack of other treatment options.

## Supplementary information

HDCT in male GCC patients - Appendix

Reproducibility checklist - HDCT in male GCC patients

## Data Availability

The data in our study result from the nationwide SWENOTECA registry as described in the method section. Restrictions apply for the availability of these data according to the national data protection legislation. Data are available from the authors with the permission of the Swedish Authority for Privacy Protection. Additional information will be available from the corresponding author upon request.
