# Supplementary material for: High-dose chemotherapy in male germ cell cancer patients—a study by the SWENOTECA group
Source: Br J Cancer. 2025 Dec 22;134(5):746–53. doi: 10.1038/s41416-025-03322-9 (PMC12905181; doi:10.1038/s41416-025-03322-9)
Supplement: Supplementary file 1 — HDCT in male GCC patients - Appendix [file 41416_2025_3322_MOESM1_ESM.docx]

**Appendix

Table S1. Number of high-dose cycles and reasons for only one cycle, by indication for high-dose chemotherapy**

|  | **Delayed marker decline, *N* = 26 (%)** | **Progression*,**  ***N* = 29 (%)** | **Relapse, *N* = 25 (%)** | **All patients**  ***N* = 80 (%)** |
| --- | --- | --- | --- | --- |
| **Number of high-dose cycles** |  |  |  |  |
| - **One cycle** | 7 (27) | 10 (34) | 7 (28) | 24 (30) |
| - **Two cycles** | 19 (73) | 19 (66) | 18 (72) | 56 (70) |
| **Reasons for receiving only one high-dose cycle** |  |  |  |  |
| - **Toxicity** | 4 (15) | 2 (7) | 4 (16) | 10 (13) |
| - **Progression** | 2 (8) | 6 (21) | 3 (12) | 11 (14) |
| - **Not enough stem cells** | 1 (4) | 1 (3) | 0 (0) | 2 (3) |
| - **Other** | 0 (0) | 1 (3) | 0 (0) | 1 (1) |

*Progression: Progression within 3 months from last chemotherapy treatment.


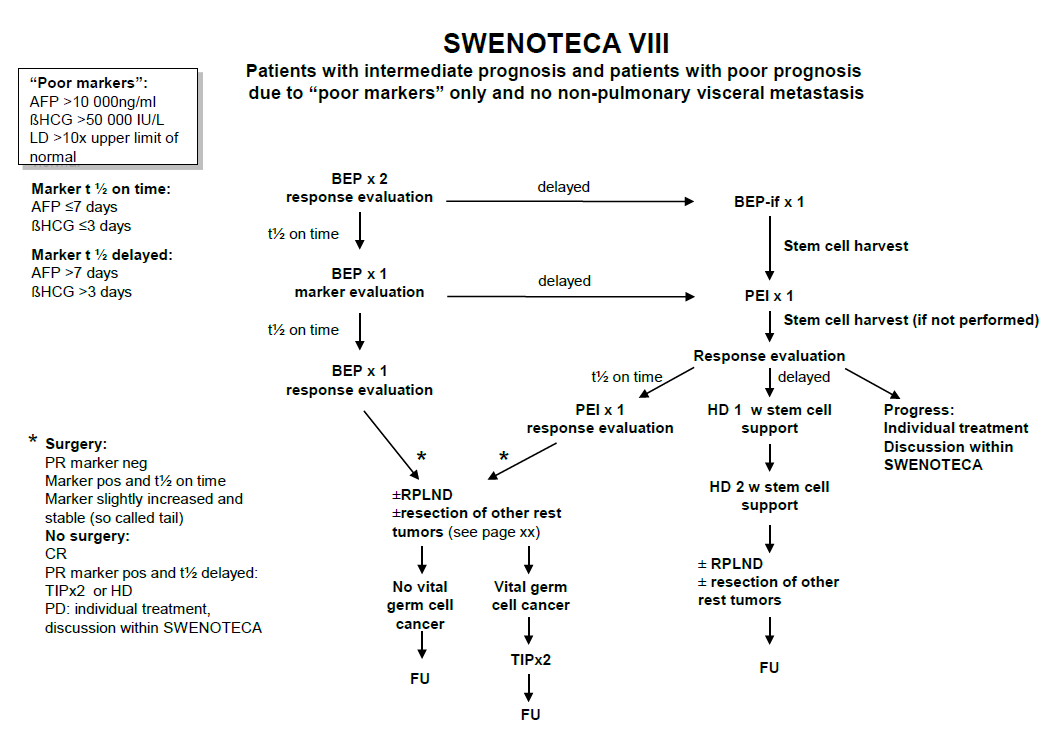
**Figure S1a.**


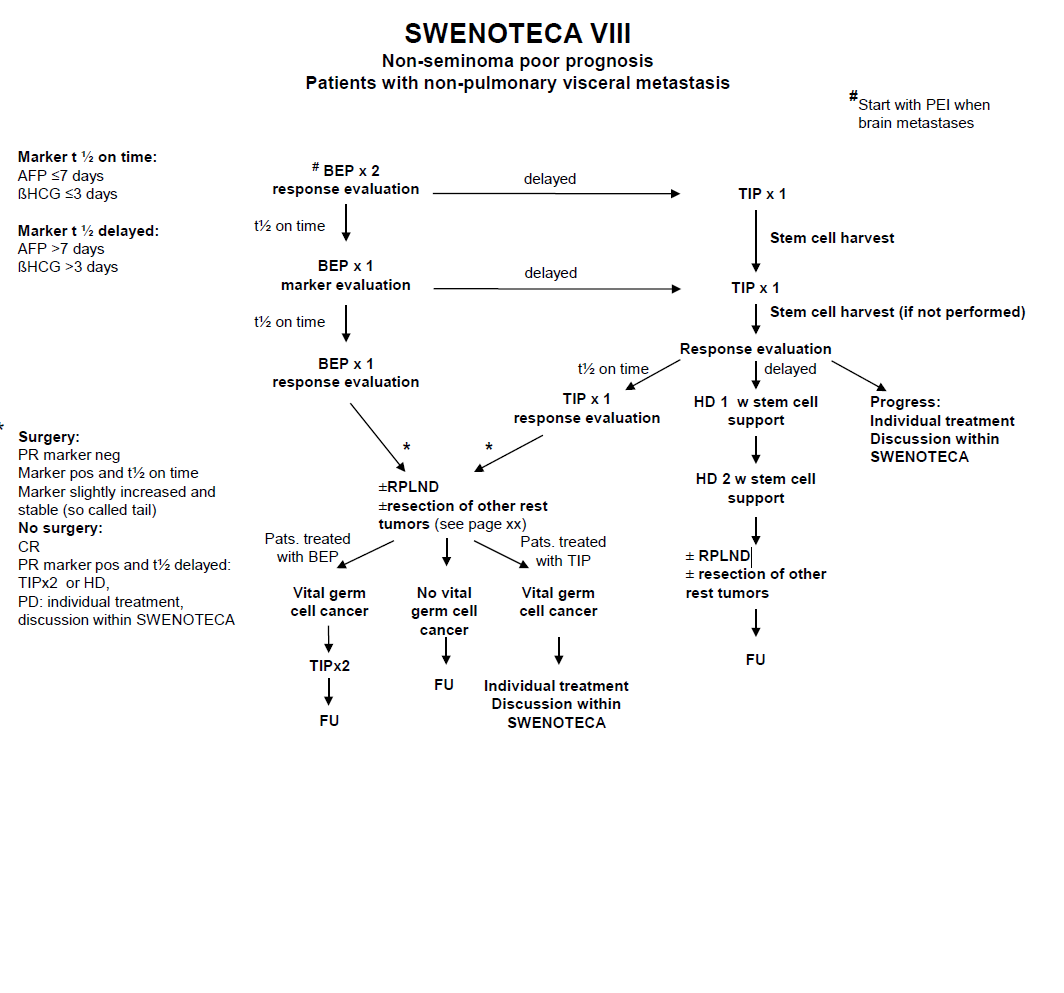
**Figure S1b.**

**Figure S1c.**

**
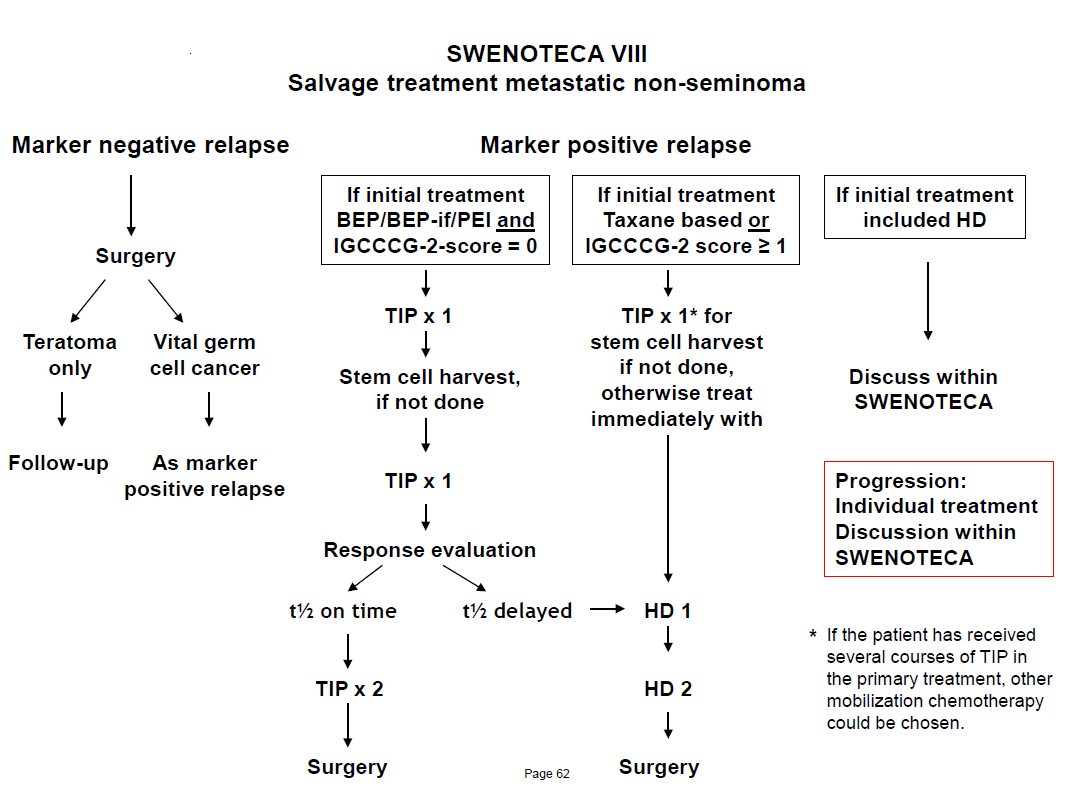
**IGCCCG-2 score: Also known as IPFSG-score

**Figure S2. Flow chart of included patients.

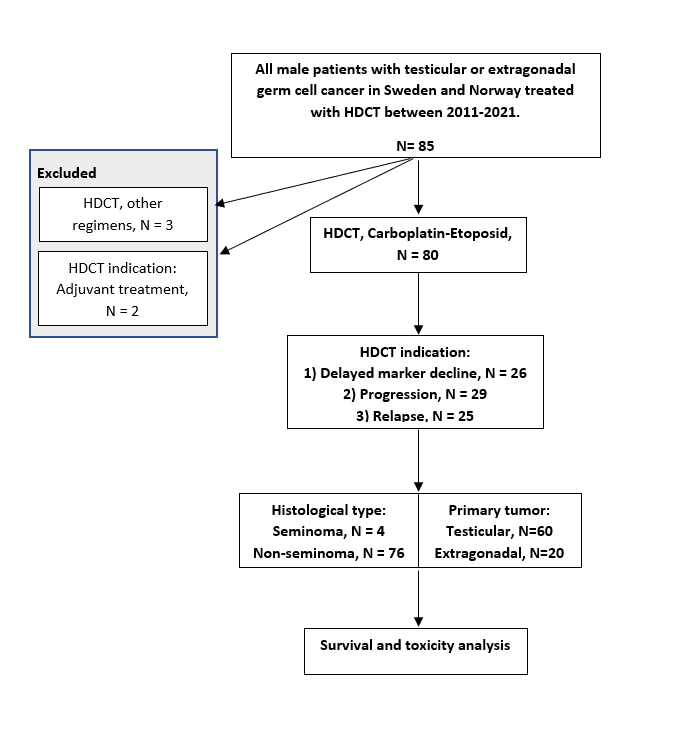
**


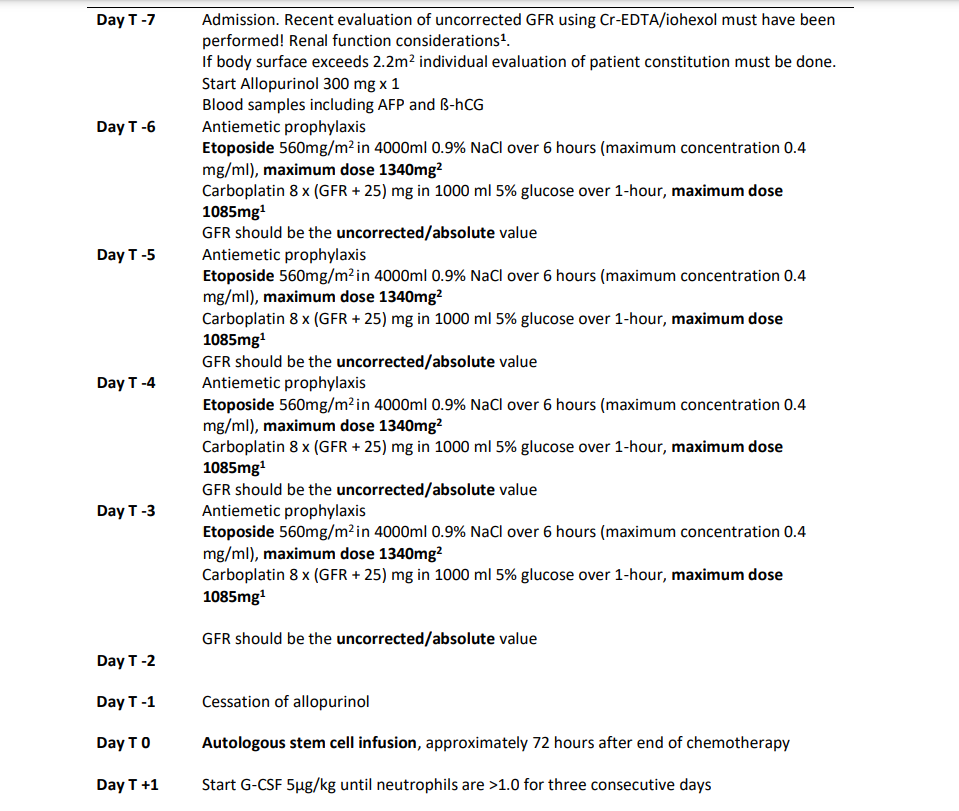
**Figure S3. HDCT regimen Carboplatin-Etoposid.**
